# Treatment of Hydrocele

**Published:** 1865-06

**Authors:** 


### MISCELLANEOUS.

TREATMENT OF HYDROCELE.—Dr. William Jollie (Gateshead-on-Tyne) recommends the following plan, which he has followed for some years, and invariably with success, even after failure with port wine, etc.:

"I tap the hydrocele by trocar and canula in the usual way, draw off the fluid, and then introduce through the canula into the cavity of the tunica vaginalis a common surgeon's probe, which has been previously coated for an inch of its length with nitrate of silver. I prepare the probe by heating the extremity to a dull, red heat in the flame of a gas-light, and placing it in a little finely-powdered nitrate of silver, and then again subjecting the probe to the heat, so as to form a smooth coating to the instrument. If your correspondent and other surgeons will make use of this method, they will, I have no doubt, quickly, effectually, and cheaply relieve their patients of a troublesome complaint."—*Lancet.*

